# Adoption is not associated with immunological and virological outcomes in children with perinatally acquired HIV infection in the Netherlands

**DOI:** 10.1371/journal.pone.0284395

**Published:** 2023-05-04

**Authors:** Malon Van Den Hof, Colette Smit, Annemarie M. C. Van Rossum, Pieter L. A. Fraaij, Tom F. W. Wolfs, Sibyl P. M. Geelen, Henriette J. Scherpbier, Elisabeth H. Schölvinck, Koen Van Aerde, Peter Reiss, Ferdinand W. N. M. Wit, Dasja Pajkrt, on behalf of the ATHENA cohort study group

**Affiliations:** 1 Paediatric Infectious Diseases, Emma Children’s Hospital, Amsterdam University Medical Centers, Location Academic Medical Center, University of Amsterdam, Amsterdam, the Netherlands; 2 HIV Monitoring Foundation, Amsterdam, the Netherlands; 3 Department of Paediatrics, Sophia Children’s Hospital, ErasmusMC, Rotterdam, the Netherlands; 4 Department of Paediatrics, Wilhelmina Children’s Hospital, Utrecht, the Netherlands; 5 Department of Paediatrics, Beatrix Children’s Hospital, University Medical Centre Groningen, Groningen, the Netherlands; 6 Department of Paediatrics, Amalia Children’s Hospital, Radboud University Medical Centre, Nijmegen, the Netherlands; 7 Department of Global Health, Amsterdam University Medical Centers, Location Academic Medical Center, University of Amsterdam and Amsterdam Institute for Global Health and Development, Amsterdam, the Netherlands; 8 Department of Internal Medicine, Division of Infectious Diseases, Amsterdam University Medical Centers, Location Academic Medical Center, University of Amsterdam Infection and Immunity Institute, Amsterdam, the Netherlands; University of Ghana College of Health Sciences, GHANA

## Abstract

**Objectives:**

To provide an overview of the demographics, treatment characteristics and long-term outcomes of children with perinatal HIV-1 infection (PHIV) living in the Netherlands (NL) and to specifically investigate whether outcomes differ by children’s adoption status.

**Design:**

A prospective population-based open cohort including children with PHIV in NL.

**Methods:**

We included children with PHIV who had entered HIV care in NL since 2007, in view of a sharp increase in the number of adopted children with PHIV since that year. We compared the proportion with virologic suppression and CD4^+^T-cell count over time between the following groups of children with PHIV: adopted and born outside NL, non-adopted born in NL, and non-adopted born outside NL, using generalized estimating equations and linear mixed effects models, respectively. To account for the variation in cohort inclusion, we analyzed data of children exposed to at least one year of antiretroviral therapy (ART).

**Results:**

We included 148 children (827.5 person-years of follow-up, 72% adopted, age at start care in NL 2.4 (0.5–5.3)). Under-18 mortality was zero. Over the years, a boosted PI-based regimen was most often prescribed. The use of integrase inhibitors increased since 2015. Non-adopted children born in NL were less likely to achieve virological suppression compared to adopted children (OR 0.66, 95%CI 0.51–0.86, *p* = 0.001), which disappeared after excluding one child with suspected treatment nonadherence (OR 0.85, 95%CI 0.57–1.25, *p* = 0.400). CD4^+^T-cell Z-score trajectories were not significantly different between groups.

**Conclusions:**

Despite considerable and increasing diversity of the population of children with PHIV in NL, geographical origin and adoption status do not seem to pose important challenges in achieving good immunological and virological outcomes.

## Introduction

As a result of successful prevention of mother-to-child transmission of human immunodeficiency virus type 1 (HIV-1) infection, the demographics of the population of children with perinatally acquired HIV infection (PHIV) has changed considerably over the last decade. In the Netherlands (NL), universal first trimester HIV-screening for pregnant women was implemented in 2004, based on an opting-out approach. Since then, vertical transmission has been greatly reduced and has become very rare [1–3]. Currently, the majority of children with PHIV living in NL were born outside NL, and were either adopted by Dutch adoptive parents or entered NL as migrants together with their biological parents [3].

Guidelines on the treatment of pediatric HIV have changed importantly over the last decade, moving from the recommendation in 2010 to immediately initiate combination antiretroviral therapy (ART) in all HIV-infected children under the age of two–irrespective of clinical disease stage and CD4^+^T-cell count–to start in all children under the age of five in 2013, and most recently (2015) to the recommendation to initiate combination ART immediately in every child living with HIV [4]. Not only has the moment of combination ART initiation changed, the preferred initial antiretroviral regimens did as well, with the most recent change being the availability of integrase strand transfer inhibitors (INSTI) for pediatric use [5].

Whether the demographic change in children with PHIV in NL comes together with a change in long-term outcomes remains unknown. Previous studies that used nationwide data from NL found an increase in the percentage of children with an undetectable HIV load over time with similar long-term immune reconstitution and virologic response to combination ART between children with PHIV born in Sub-Saharan Africa and those born in NL [6, 7]. However, at the time of that analysis, the number of adopted children was still relatively small. Internationally adopted children are often exposed to adverse early life experiences, making them vulnerable to behavior problems which may potentially lead to poorer HIV- and treatment- related clinical outcomes [8].

To extend previous findings, this study aims to provide an overview of the demographic characteristics, prescribed antiretroviral therapy, immunological and virological outcomes of children with PHIV in care in NL and to specifically investigate whether long-term immunological and virological outcomes differ by children’s adoption status. Knowledge on potential differences may lead to optimization of care.

## Methods

### Study design and population

We used fully anonymized data from the AIDS THerapy Evaluation in the NetherlAnds (ATHENA) cohort, a prospective population-based cohort that monitors the clinical outcomes of all HIV-positive people in HIV care in NL since the introduction of combination ART in 1996. The ATHENA cohort, managed by the Dutch HIV monitoring Foundation, collects clinical data from medical records from all adults and children in HIV care in NL (2% opt-out) [3, 9]. This work complies with the principles of the Declaration of Helsinki. We included data from 2007 onwards, ensuring to capture the sharp increase in the number of adopted children with PHIV since that year [3]. We included children with PHIV who had entered care in NL since 2007 and received care in one of the Dutch pediatric HIV treatment centers (Amsterdam University Medical Centers, location Academic Medical Center; University Medical Center Utrecht; Erasmus Medical Center, Rotterdam; Radboud University Medical Center and University Medical Center, Groningen). We included data since 2007 in view of a sharp increase in the number of adopted HIV-1 infected children since that year. This increase is a result of a rise in the number of special need adoptions, i.e. adoption due to specific factors or conditions, such as HIV infection, as a consequence of fewer healthy children being available for adoption. We assumed PHIV infection when reported as such by the treating pediatrician or the adoption documents. When not explicitly reported, we presumed perinatal transmission in children in whom HIV infection had been diagnosed before the age of five. We used data from participants until eighteen years of age, loss of follow-up, death or database closure on 31th December 2017. At the moment of database closure, for every child in care, at least one visit in 2017 had been recorded.

### Outcome variables

We report on the following outcome measures: all-cause mortality, ART prescription, Centers for Disease Control and Prevention (CDC)-defined opportunistic events [10], HIV-RNA viral load (VL) and CD4^+^T-cell Z-score. Follow-up started at date of registration with the Dutch HIV monitoring foundation and continued to the censoring date.

We investigated trends in ART prescriptions among all children with PHIV by using July 1^st^ as reference date for each year between 2007 and 2017. We categorized ART regimens as follows: no ART, monotherapy (one single antiretroviral drug), INSTI + nucleos(t)ide reverse transcriptase inhibitor (NRTI) backbone, INSTI + protease inhibitor (PI) with or without a NRTI backbone, PI with backbone, non-nucleoside reverse transcriptase inhibitor (NNRTI) + NRTI backbone, and the category ‘other’. We defined NRTI backbone as the use of two or more NRTI’s. We defined a period of no ART between two regimens as a period of 31 consecutive days with no ART prescription.

We investigated opportunistic events (categorized according to the CDC classification, as B or C type events), virological and immunological outcomes in children with PHIV who had an ART prescription for a minimum of one year and who had at least had six months of follow-up, to select an adequately treated study group and to adjust for different time calendar periods of entering HIV care. To investigate virological and immunological outcomes, we included measures that were closest to the previous measure with an interval of six months. We dichotomized HIV VL using a cut-off of 200c/mL to differentiate between undetectable HIV VL including viral blips, and higher levels of viraemia. As CD4^+^T-cell counts tend to be higher in children compared to adults [11], we investigated immunological outcome by standardizing absolute CD4^+^T-cell counts to Z-scores to adjust for these age-related differences. To do this, we subtracted the age-related reference value (categorized in: under the age of 9 months, 9–15 months, 15–24 months, 2-5y, 5-10y, 10-16y and 16-18y) at the time of CD4^+^T-cell measurement from the absolute CD4^+^T-cell count and we divided this by the age-related standard deviation (SD) [12].

### Exposure variables

We categorized children with PHIV according to their adoption status (yes or no), and categorized non-adopted children into born in or outside the Netherlands, as we assumed that sociodemographic variables would differ between these non-adopted children. This resulted in the following groups: 1) adopted children, 2) non-adopted children born in NL, and 3) non-adopted children and born outside NL. Throughout this paper, “adopted” means “adopted born outside the Netherlands”. We defined age at time of adoption as the age of registration with the ATHENA database, as newly adopted children with PHIV are assessed by a pediatrician shortly after arrival in NL.

### Potential confounders

We investigated age at ART initiation and socioeconomic status (SES) as potential confounders. We categorized SES as ‘very wealthy’, ‘wealthy’, ‘average’, ‘less-favored’ and ‘deprived’, based on the four-digit postal code of residence at the time of registration with the HIV Monitoring Foundation, as described by the Netherlands Institute of Social Research (SCP) [13]. Statistics Netherlands (www.cbs.nl) makes available data on the average SES in each four-digit postcode area by aggregating the available relevant information of all residents.

### Statistical analysis

We generated descriptive statistics and compared sociodemographic characteristics, virological and immunological outcomes between the three groups. To test whether distributions of variables differed significantly between groups, we used the one-way ANOVA for normally distributed variables and the Kruskal-Wallis test for non-normally distributed data.

Between 2007 and 2017, we assessed the relationship between calendar year and the proportion of children with PHIV with HIV VL below 200c/mL, and between calendar year and CD4^+^T-cell Z-score by Generalized Estimating Equations (GEE) logistic regression (using an exchangeable correlation structure) and linear mixed effects models, respectively. The odds ratio (OR) of the model represented the ratio between the odds for being non-adopted and born in NL compared to being adopted, and for being non-adopted and born outside NL compared to being adopted. In the linear mixed effects model, we included CD4^+^T-cell Z-score as a continuous variable and we included a random intercept to account for intra-subject correlation. In both models, we considered a covariate a significant confounder if the introduction into the model caused a change in the regression coefficient of calendar year by more than 10 percent. To investigate whether there are differences between groups over time, we included a group-by-time interaction term.

We defined a missing plasma HIV VL or CD4^+^T-cell Z-score as the absence of a measure of these outcomes in the period from three months prior to three months later the intended date of visit at interval of six months. We handled missing data in these variables using the last observation carried forward approach. In case the date of initiation of first-line ART was missing, we imputed this with the date of initiation of the earliest recorded ART regimen. In case a date of initiation of the earliest recorded ART regimen was missing, we imputed this with the date of entry into HIV care.

Post-hoc, we performed a sensitivity analysis in which we repeated the HIV VL and CD4^+^T-cell Z-score models, after excluding data from one non-adopted child born outside NL with persistently high HIV VL. This was done to investigate whether this child was driving the significantly lower odds ratio (OR) of having HIV VL below 200c/mL compared to children with PHIV who had been adopted. Statistical analyses were performed using RStudio, version 1.1.453 [14]. A two-sided *p*<0.05 was considered statistically significant.

## Results

### Sociodemographic characteristics

Of the total 318 children with PHIV ever registered with the Dutch HIV monitoring foundation, 148 children entered care since 2007 and were included in the analysis (S1 Fig). Table 1 shows their relevant characteristics, stratified according to geographical origin and adoption status. Four children (3%) moved abroad during follow-up, which resulted in the discontinuation of the registration of their data by the Dutch HIV monitoring foundation. Children with PHIV had a mean follow-up time of 5.6 years (SD 2.6, range 0.1–10.1), for a total of 827.5 person years of follow-up.

**Table 1:** Characteristics of children with Perinatally acquired Human Immunodeficiency Virus infection (PHIV) in the Netherlands (NL) newly entering care since 2007, stratified according to geographical origin and adoption status. ^a^ *p*-value of the comparison between three groups: adopted, non-adopted born in NL and non-adopted born outside NL. Abbreviations: ART, antiretroviral therapy; Latin America represents: Brazil, French Guiana, Nicaragua, Suriname and Venezuela. ‘Other’ represents: Canada, China, Morocco, Poland, Saudi Arabia, Ukraine and the USA. Southeast Asia represents: Indonesia, India and Cambodia. Western Europe represents: Belgium, Germany, Spain, France and Portugal.

	Total group (n = 148)		Adopted (n = 106)		Non-adopted				
					Born in NL (n = 10)		Born outside NL (n = 32)		
	N		N		N		N		P-value^a^
**Male sex, N (%)**	148	64 (43%)	106	47 (44%)	10	5 (55%)	32	12 (38%)	0.718
**Region of origin, N (%)**	148		106		10		32		<0.001
Sub-Saharan Africa		119 (80%)		94 (89%)		0 (0%)		25 (78%)	
The Netherlands		10 (7%)		0 (0%)		10 (100%)		0 (0%)	
Latin America		4 (3%)		4 (4%)		0 (0%)		0 (0%)	
Western Europe		3 (2%)		1 (1%)		0 (0%)		2 (6%)	
Southeast Asia		4 (3%)		1 (1%)		0 (0%)		3 (9%)	
Other		8 (5%)		6 (6%)		0 (0%)		2 (6%)	
**Country of origin (biological) Mother, N (%)**	88		33		10		19		<0.001
Sub-Saharan Africa		72 (82%)		30 (91%)		5 (50%)		15 (79%)	
The Netherlands		3 (3%)		0 (0%)		3 (30%)		2 (11%)	
Latin America		1 (1%)		0 (0%)		0 (0%)		1 (5%)	
Western Europe		1 (1%)		0 (0%)		0 (0%)		0 (0%)	
Southeast Asia		4 (5%)		1 (3%)		0 (0%)		0 (0%)	
Other		7 (8%)		2 (6%)		2 (20%)		1 (5%)	
**Socioeconomic status at registration, N (%)**	144		103		10		31		<0.001
Very wealthy		7 (5%)		6 (6%)		0 (0%)		1 (3%)	
Wealthy		39 (27%)		33 (32%)		0 (0%)		6 (19%)	
Average		47 (33%)		36 (35%)		1 (10%)		10 (32%)	
Less-favored		31 (22%)		21 (20%)		2 (20%)		8 (26%)	
Deprived		20 (14%)		7 (7%)		7 (70%)		6 (19%)	
Age at start care in NL (years)	148	2.4 (0.5–5.3)	106	1.8 (0.4–3.1)	10	0.3 (0.1–1.6)	32	7.5 (5.2–9.3)	<0.001
Age at HIV diagnosis (years)	148	0.8 (0.2–2.9)	106	0.4 (0.1–1.7)	10	0.3 (0.1–1.6)	32	5.8 (2.7–8.5)	<0.001
Time between diagnosis and start care in NL (years)	148	0.3 (0.0–1.5)	106	0.4 (0.0–1.6)	10	0.0 (0.0–0.0)	32	0.0 (0.0–2.2)	<0.001
Age at ART initiation (years)	136	1.5 (0.4–4.1)	98	0.9 (0.4–2.4)	10	0.3 (0.1–1.7)	28	8.2 (4.5–11.7)	<0.001
Time between HIV diagnosis and ART initiation (months)	136	2.7 (0.6–10.6)	98	2.3 (0.8–7.1)	10	0.5 (0.2–0.8)	28	7.6 (1.1–29.6)	0.009
HIV viral load at start ART (log c/ml)	66	5.3 (1.0)	38	5.5 (1.1)	8	5.3 (1.4)	20	4.8 (0.5)	0.045
CD4^+^T-cell Z-score at start ART	75	-0.9 (-1.3 to -0.4)	49	-0.9 (-1.2 to -0.3)	7	-1.0 (-1.4 to -0.5)	19	-0.8 (-1.3 to -0.7)	0.748
Pretreated (mono- or dual therapy)	142	6 (4%)	104	6 (6%)	10	0 (0%)	28	0 (0%)	0.321

The majority of children (n = 106, 72%) had been adopted by Dutch adoptive parents and had been born outside NL. All children except two (1.9%), received their HIV diagnosis prior to adoption. One child was diagnosed with HIV in the same month of entering care in the Netherlands, another child received an HIV diagnosis 11 months after entering care. Ten non-adopted children (7%) were born in NL and 32 non-adopted children (22%) were born outside NL. children with PHIV born outside NL were mainly born in sub-Saharan Africa (SSA). The majority of children with PHIV who had been born in NL (n = 5, 50%) were born to mothers who originated from SSA. The distribution of SES differed between groups, with higher SES in those who had been adopted and were born outside NL compared to both non-adopted groups.

Concerning HIV- and ART related characteristics, age at HIV diagnosis and age at ART initation were significantly different between groups (*p*<0.001), with the oldest age at HIV diagnosis and ART initiation in the non-adopted group born outside NL (median age at HIV diagnosis 5.8 years, interquartile range [IQR] 2.7–8.5; median age at ART initiation 8.2 years, IQR 4.5–11.7). In this group, the interval between HIV diagnosis and the initiation of ART was also the longest, with a median of 7.6 months (IQR 1.1–29.6). Only a small number of children (n = 6, 4%) who newly entered care since 2007 had a history of mono- or dual therapy treatment prior to initiating ART.

### Mortality

Between 2007 and 2017, all-cause mortality rate in children with PHIV who entered care since 2007 in NL was zero.

### Antiretroviral therapy

**Fig 1:**
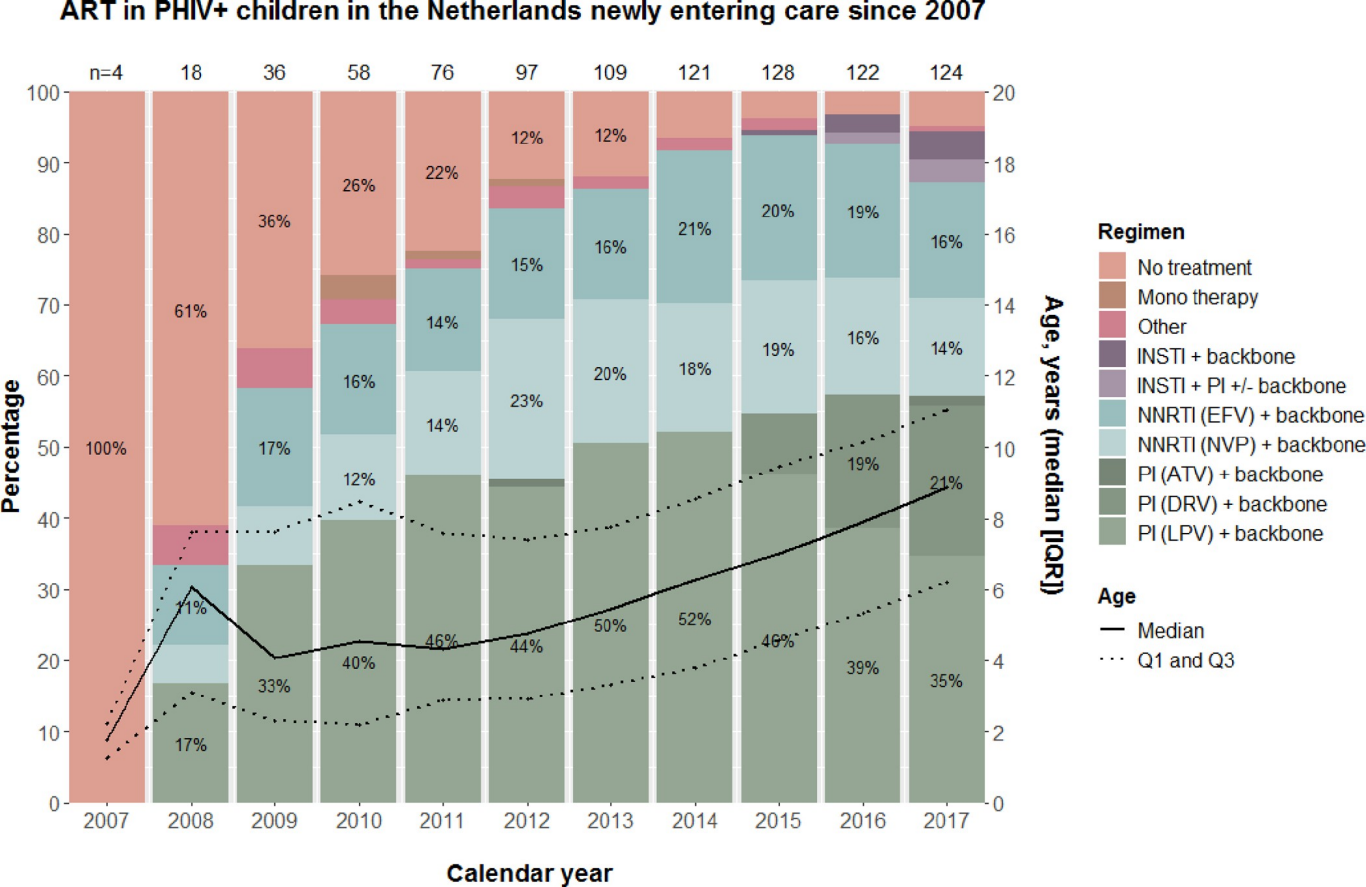
Antiretroviral therapy use and composition in children with perinatally acquired HIV infection (PHIV) in the Netherlands (NL) per calendar year. Figure shows data of children with PHIV who entered HIV care since 2007. Dual axis chart shows 1) the percentage of children on ART and using a particular regimen (left y-axis) represented by different colored stacked bars, and 2) the median age (right y-axis, solid black line) and interquartile range (dotted black lines) of the cohort of children with PHIV in care in each calendar year. The number of children in care in each calendar year are provided on top of each bar. ‘Mono’ represents NNRTI and PI monotherapy. ‘Other’ represents regimens without a NRTI backbone (1 NRTI + 1 NNRTI + 1 PI), or NRTI-only regimens (dual or triple NRTI therapy). Abbreviations: INSTI, integrase strand transfer inhibitors; NRTI, nucleos(t)ide reverse transcriptase inhibitor; NNRTI, non-nucleoside reverse transcriptase inhibitor; PI, protease inhibitor; EFV, efavirenz; NVP, nevirapine; ATV, atazanavir; DRV, darunavir; LPV, lopinavir; n, number; Q1, first quartile; Q3, third quartile.

Fig 1 shows the trend in prescribed antiretroviral therapy. The majority of children have been prescribed a boosted PI-containing regimen–most commonly containing ritonavir-boosted lopinavir–combined with a dual or triple NRTI backbone. We observed an increase in the proportion of children using darunavir-containing regimen since 2015, which coincided with an increase in age of the cohort. Between 2007 and 2017, the percentage of NNRTI prescriptions declined slightly over time and we observed the introduction of INSTI prescriptions since 2012, either prescribed with a NRTI backbone, with a boosted PI, or with both a boosted-PI and NRTI backbone. Of all INSTI prescriptions (n = 15), 53% received dolutegravir (n = 8), 40% raltegravir (n = 6) and 7% elvitegravir (n = 1). Almost all PI prescriptions included ritonavir as booster (n = 442), only a few had been prescribed cobicistat (n = 3). One child received cobicistat-boosted elvitegravir. At time of database closure in December 2017, six children (4%) were not using ART. Three of them had entered care in 2017, with their pediatricians reporting a good clinical condition with very low HIV VL and high CD4^+^T-cell counts as reason for not having started ART in two of these children.

**Fig 2:**
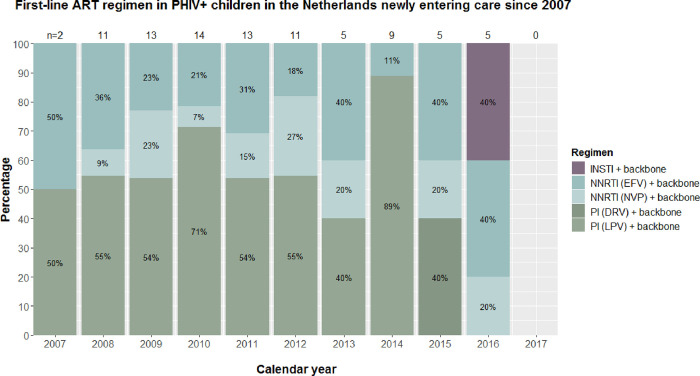
Initial antiretroviral therapy started in children with perinatally acquired HIV infection(PHIV) in the Netherlands (NL) per calendar year. Figure shows the data of PHIV children who entered HIV care in the Netherlands since 2007. Abbreviations: DRV, darunavir; EFV, efavirenz; INSTI, integrase strand transfer inhibitors; LPV, lopinavir; NVP, nevirapine; NNRTI, non-nucleoside reverse transcriptase inhibitor; n, number; PI, protease inhibitor.

Fig 2 shows the trend in initial ART regimens started between 2007 and 2017. A total of 88 children with PHIV (59%) initiated first-line ART during this period, the majority with a ritonavir-boosted PI-based regimen, followed by a NNRTI-based regimen. In 2016, we observed the first prescription of INSTI-based regimens, consisting of dolutegravir, as first-line therapy, which replaced the ritonavir-boosted PI-based regimens as first-line therapy.

There was a significant difference in the number of NNRTI-based regimen prescriptions across groups (*p*<0.001). A NNRTI-based regimen was prescribed in 43% of the non-adopted children with PHIV born outside NL, as compared to in 33% of the adopted group born outside NL and in 18% the non-adopted group born in NL. In contrast, although a boosted PI-based regimen was prescribed more frequently in adopted children with PHIV born outside NL (56%) and non-adopted children with PHIV born in NL (70%) compared to non-adopted children born outside NL (24%), this was not statistically significant (*p* = 0.054).

### Opportunistic events (categorized according to the CDC classification)

Since 2007, 37 opportunistic events occurred in 21 children with PHIV, prescribed ART for a minimum of one year, and 389.7 person-years of follow-up in adopted children born outside NL, 45.5 person-years in non-adopted children born in NL and 96.6 person-years in non-adopted children born outside NL. This corresponds to an overall incidence rate of 7.0 opportunistic events per 100 person-years, with a rate of 8.2 per 100 person-years in adopted children born outside NL, 2.1 per 100 person-years in non-adopted born in NL and 4.0 per 100-person-years in non-adopted born outside NL (not being statistically different between groups). The majority (65%) of occurring opportunistic events was classified as CDC-A (n = 24): hepatomegaly (n = 5), lymphadenopathy (n = 6), dermatitis (n = 6), parotitis (n = 1) and recurrent or persistent respiratory infection (n = 6). Eleven events (30%) were categorized as CDC-B events: oropharyngeal candidiasis (n = 1), diarrhea of unknown origin for at least one month (n = 1), multidermatomal herpes zoster infection (n = 1), anemia (hemoglobin 9 mg/dL for at least 30 days) (n = 1), bacterial meningitis/pneumonia or sepsis (n = 3), neutropenia (neutrophils <1000/mm^3^ for at least 30 days) (n = 3), oral candidiasis for at least two months (n = 1). Two events (5%) were categorized as AIDS defining (CDC-C): extrapulmonary tuberculosis (n = 1), or multiple/recurrent bacterial infections (n = 1).

### Long-term virologic response to combination antiretroviral therapy

**Fig 3:**
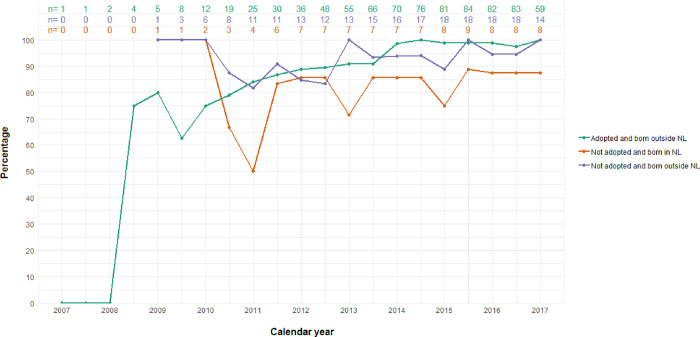
Percentage of PHIV children with virological suppression over time. Figure shows the data of PHIV children who entered HIV care in the Netherlands since 2007 and who have been prescribed at least one year of antiretroviral therapy (ART). Virological suppression was defined as HIV viral load below 200c/mL. Different groups of geographical origin and adoption status are represented by green (adopted and born outside the Netherlands [NL]), red (not adopted and born in NL), and purple (not adopted and born outside NL).

In 129 children, 64 measures were missing out of 1192 HIV VL observations (5%). This included 58 measures out of 873 observations (6%) in adopted children born outside NL, 0 measures out of 71 observations (0%) in the non-adopted children born in NL, and 6 measures out of 248 observations (2%) in the non-adopted children born outside NL. Fig 3 shows the proportion of children with PHIV with HIV VL below 200c/mL per calendar year. The odds of having HIV VL below 200c/mL increased significantly by calendar year (odds 1.48, 95%CI 1.28–1.73, *p*<0.001), without age acting as a confounder in this relationship. Non-adopted children born in NL were less likely to achieve HIV RNA below 200c/mL compared to adopted children born outside NL (OR 0.66, 95%CI 0.51–0.86, *p* = 0.001). After excluding the one child with persistent high level viraemia despite ART prescription from the model, this relationship was lost (OR 0.85, 95%CI 0.57–1.25, *p* = 0.400).

### Long-term immunologic response to combination antiretroviral therapy

**Fig 4:**
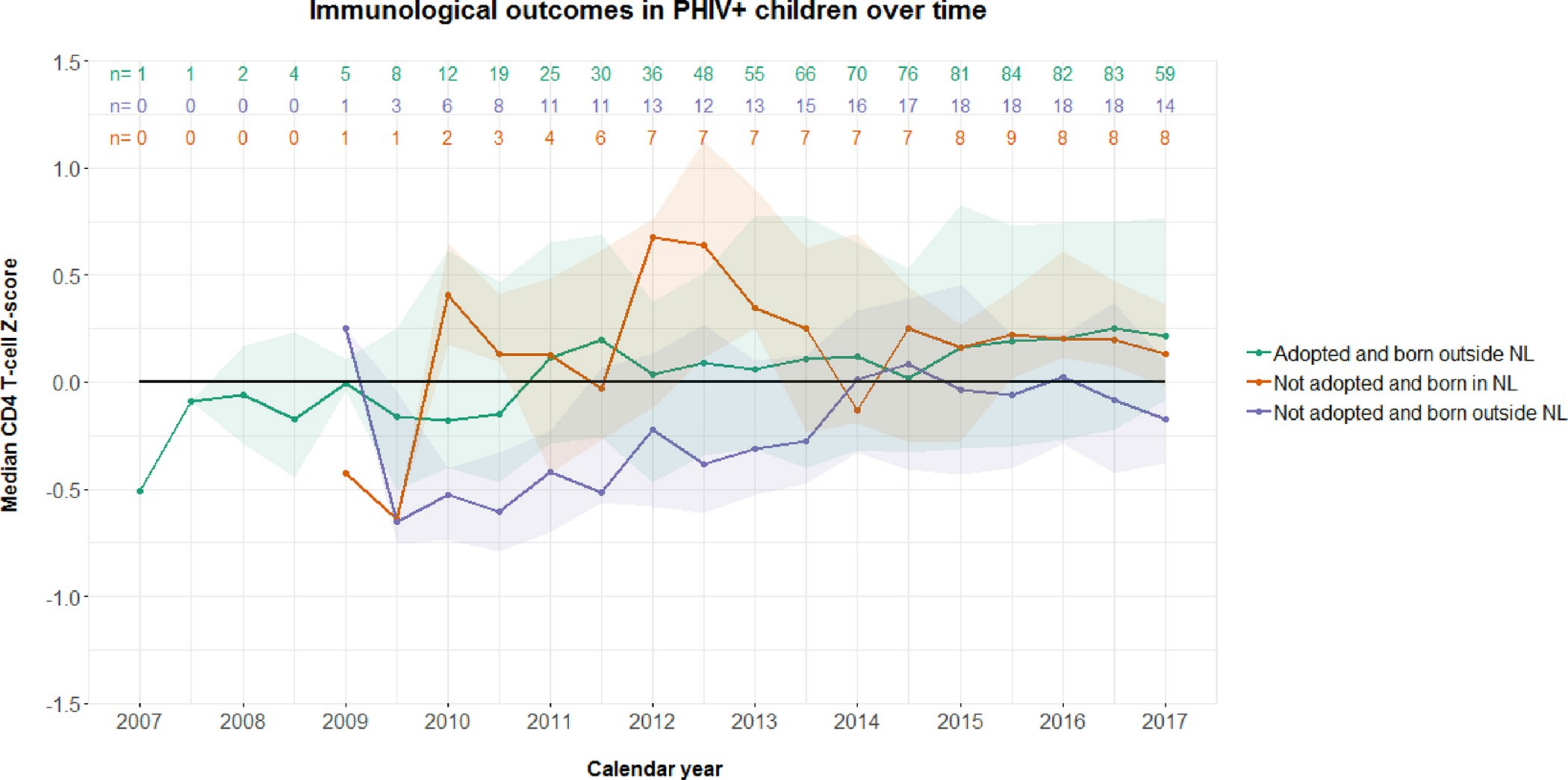
CD4^+^T-cell Z-score among children with perinatally acquired HIV infection (PHIV) in the Netherlands (NL) over time. Figure shows the data of PHIV children who entered HIV care since 2007 and who have been prescribed at least one year of antiretroviral therapy (ART). We generated Z-scores out of absolute CD4^+^T-cell counts using age-related reference values. Different groups of geographical origin and adoption status are represented by green (adopted and born outside the Netherlands [NL]), red (not adopted and born in NL), and purple (not adopted and born outside NL). The shaded areas in the corresponding colors represent the interquartile range.

286 measures were missing out of 1192 CD4^+^T-cell observations (24%) in 129 children: 233 out of 886 observations (28%) in adopted children born outside NL, 18 out of 101 observations (18%) in non-adopted children born in NL, and 35 out of 218 observations (16%) in non-adopted children born outside NL. The trajectories of CD4^+^T-cell Z-score were similar between groups and between -1 and +1SD of the norm (Interaction calendar year*Origin non-adopted and born in NL compared to adopted born outside NL: -0.055, 95%CI -0.125–0.016, *p* = 0.128; calendar year*Origin non-adopted and born outside NL: 0.024, 95%CI -0.024–0.072, *p* = 0.322) (Fig 4).

## Discussion

In this national population-based cohort study, we demonstrate that long-term virological and immunological outcomes in children with PHIV in NL do not differ by adoption status and geographical origin. Between 2007 and 2017, under-18 mortality was zero and the CDC-event rate was low. During this ten year period, ritonavir-boosted PI-based regimens were prescribed most commonly, overall and as initial therapy. Since 2016, there is clear evidence that INSTI-based regimens are replacing PI-based regimens as first-line therapy.

Due to successful mother-to-child prevention of HIV transmission in NL, a demographical shift has occurred in the population of children with PHIV over the last decade. Where a previous study of this kind–including data up to 2013–showed that children with PHIV were most often born in NL [6], nowadays the majority of children with PHIV newly entering care have been adopted by Dutch parents. This shift has also been acknowledged in other countries [15, 16].

The population of children with PHIV who were registered in NL since 2007 as a result also demonstrates considerable diversity in demographic and HIV- and treatment-related characteristics. Household SES, for example, varies widely in the population of children with PHIV in NL, with a relatively high SES in adopted children, potentially explained by the financial barriers adoptive parents need to overcome. Despite this diversity, long-term virological and immunological outcomes did not differ between groups. Between 2007 and 2017, children with PHIV showed an increasing likelihood of achieving viral suppression. This might be explained by an improvement in treatment adherence due to the availability of simplified ART regimens, as well as more potent and better-tolerated antiretroviral therapy.

Initially, our results suggested that non-adopted children born in the Netherlands were less likely to achieve suppressed viraemia over time. However, after excluding the one child on ART with persistent high viraemia, likely due to treatment nonadherence, this significant difference completely disappeared. The CD4^+^T-cell Z-score over time also did not significantly differ between children’s geographical origin and adoption status, with all measures being within -1 and +1SD. In children with PHIV in general, one needs to be aware of the potential occurrence of survivor bias, i.e. a selection effect in which survivors are more likely to enter adoption procedures or migrate, and consequently may have been disproportionately represented in the study group compared to the population.

Although the observed zero under-18 mortality in children with PHIV in NL is very encouraging, adolescents and young adults with HIV are known to be a vulnerable group [17], with poorer retention in care, higher rates of (temporary) treatment interruption, higher rates of virological failure and higher rates of mortality compared to HIV-infected children and adults globally [18]. Therefore, continuation of adequate care after transition to adult care is highly important and longer follow-up of this study is recommended.

Therapeutic options in children living with HIV has evolved over the years, particularly in the most recent years. In this study, we observed the prescription of integrase inhibitors among children since 2012, and as part of first-line therapy since 2016. Although recommended as first-line therapy in adults living with HIV due to its virologic efficacy, favorable toxicity profile and lack of drug interactions, an INSTI-based regimen is not yet recommended as first-line therapy in children in Europe [5]. Presently, raltegravir, dolutegravir and elvitegravir (in combination with cobicistat, emtricitabine and tenofovir alafenamide fumerate) have been authorized by the European Medicines Agency for use in the European Union in children in the following pediatric populations: raltegravir in children over four weeks of age (since June 2014), dolutegravir in those above six years of age and at least 15 kilograms (since December 2016), and elvitegravir in children older than twelve years of age and at least 25 kilograms (since November 2017) [19–21]. Bictegravir is currently under investigation for use in children.

Boosted-PI, most commonly including ritonavir-boosted lopinavir, and NNRTI-based regimens, including either efavirenz or nevirapine, were the most commonly prescribed regimens in PHIV in NL, consistent with the current guidelines for first-line ART regimens in children [22]. A ritonavir-boosted PI-based regimen is among the recommended preferred first-line ART in PHIV children, with ritonavir-boosted lopinavir recommended for children younger than six years old, ritonavir-boosted atazanavir for children 6–12 years old and ritonavir-boosted atazanavir or ritonavir-boosted darunavir for children above twelve years old [22]. In this study, we observed only a few prescriptions of atazanavir, potentially because of the concern of scleral icterus as a result of atazanavir-associated hyperbilirubinemia [23].

According to the current data, both adopted and non-adopted children born outside NL reached the goal of at least 90% viral suppression, as part of the 90-90-90 target [24]. These targets indicate for 90% of all people living with HIV to know their HIV status, 90% of those to receive sustained ART, and 90% of those receiving ART to achieve suppressed viraemia. Concerning the third 90 there remains room for improvement for non-adopted children born in NL (87.5%).

The findings of this study are subject to some limitations. First, the small sample size of non-adopted children in this study limits our power to detect potentially relevant differences between groups. Second, we standardized CD4^+^T-cell counts in this analysis based on age. However, other factors, such as children’s ethnicity might play a role, we did not take into consideration [12]. We had no data on treatment adherence, and therefore we were unable to take this into account. Although population studies commonly use post-code as a proxy for household SES, we acknowledge the imprecision of this indirect measure. This study focused on record-based outcomes and we did not investigate behavioral health outcomes or patient-reported outcomes. We acknowledge the importance of these outcomes in the population of adopted children and recommend future research to investigate these.

In conclusion, in this nationwide study of PHIV children living with HIV in NL with 10 years follow-up of data, we found that long-term virological and immunological outcomes appear to be independent of geographical origin and adoption status. These findings demonstrate that, despite the considerable and increasing diversity of the population of children living with PHIV in NL, this diversity does not appear to pose important challenges in achieving good outcomes of care. To improve the health of children globally, one should focus on prevention of mother-to-child transmission of HIV—as this strategy has been shown to be highly effective—as well as prompt diagnosis and initiation of life-long treatment with effective and well-tolerated regimen in case prevention efforts have failed.

## Supporting information

S1 Fig Flow-chart. Flow chart of inclusion and exclusion of perinatally HIV-infected children who newly entered care since 2007. The numbers represent the number of children included for each individual outcome. Abbreviations: ART, antiretroviral therapy; CDC, Center for Disease Control and prevention category.
(DOCX)
