# Supplementary material for: Adoption is not associated with immunological and virological outcomes in children with perinatally acquired HIV infection in the Netherlands
Source: PLoS One. 2023 May 4;18(5):e0284395. doi: 10.1371/journal.pone.0284395 (PMC10159147; doi:10.1371/journal.pone.0284395)
Supplement: S1 Fig — Flow chart of inclusion and exclusion of perinatally HIV-infected children who newly entered care since 2007. The numbers represent the number of children included for each individual outcome. Abbreviations: ART, antiretroviral therapy; CDC, Center for Disease Control and prevention category. (DOCX) [file pone.0284395.s001.docx]

**Supporting information**

PHIV+ children who newly entered care since 2007 in the Netherlands

(n=148)

Outcome:

Mortality

(n=148)

ART for a minimum of one year AND >6 months of follow up

Outcome:

- Prescribed ART (n=148)

- First line ART (n=88)

Outcome:

HIV VL

(n=129)

Outcome:

CD4^+^ T-cell Z-score (n=129)

Outcome:

CDC

(n=129)

**Figure S1: Flow-chart.** Flow chart of inclusion and exclusion of perinatally HIV-infected children who newly entered care since 2007. The numbers represent the number of children included for each individual outcome. Abbreviations: ART, antiretroviral therapy; CDC, Center for Disease Control and prevention category.
